# Driving a GaAs film to a large-gap topological insulator by tensile strain

**DOI:** 10.1038/srep08441

**Published:** 2015-02-13

**Authors:** Mingwen Zhao, Xin Chen, Linyang Li, Xiaoming Zhang

**Affiliations:** 1 School of Physics and State Key Laboratory of Crystal Materials, Shandong University, Jinan, Shandong, 250100, China

## Abstract

Search for materials with a large nontrivial band gap is quite crucial for the realization of the devices using quantum spin Hall (QSH) effects. From first-principles calculations combined with a tight-binding (TB) model, we demonstrate that a trivial GaAs film with atomic thickness can be driven to a topological insulator with a sizable band gap by tensile strain. The strain-induced band inversion is responsible for the electronic structure transition. The nontrivial band gap due to spin-orbital coupling (SOC) is about 257 meV, sufficiently larger for the realization of QSH states at room temperature. This work suggests a possible route to the fabrication of QSH-based devices using the well-developed GaAs technology.

Topological insulators (TIs), also known as quantum spin Hall (QSH) insulators, are new quantum states of matter with a bulk electronic band gap due to spin-orbital coupling (SOC) and gapless surface or edge states protected by time-reversal symmetry^1^,^2^,^3^,^4^. The surface or edge states characterized by Dirac-cone-like linear energy dispersion support the transport of charge and spin on the surfaces or at the edges^5^,^6^. For two-dimensional (2D) TIs, the edge states are more robust against backscattering than the surface states in three-dimensional (3D) TIs, which are quite promising for the realization of conducting channels without dissipation. A large SOC band gap is a critical factor for the realization of such QSH-based devices. This has motivated an intensive search for large-gap 2D TI materials.

Graphene was firstly proposed as a 2D TI to realize QSH states^1^. Unfortunately, the SOC gap in graphene is unobservably small (~10^−3^ meV)^7^,^8^,^9^,^10^, which limits the operating regime to unrealistically low temperatures. The SOC strength in the graphene analogs of other carbon family elements, such as silicene, germanene, and stanene, is enhanced due to the buckling configurations and heavy atomic masses^11^,^12^. The SOC gap in germanene ~24 meV is comparable to the thermal energy at room temperature (26 meV). Silicene films have been synthesized, but they interact strongly with substrates^13^,^14^,^15^. The electronic states of silicene and substrate materials mix in the region near the Fermi level, making the realization of QSH states difficult. Moreover, the substrate may destroy the nontrivial topologies of silicene and germanene by introducing a trivial gap at the Dirac points. The instability of these graphene analogs due to the dangling bonds of the sp^3^-hybridized atoms also impedes their applications under realistic conditions.

Apart from the graphene analogs of group IV elements, the buckled honeycomb lattices of the binary compositions of group III elements and bismuth have been proposed as a new class of 2D TIs with large SOC gaps^16^. However, they face the same problem in stability as silicene and germanene due to the dangling bonds on the surfaces. A promising solution for silicene and germanene is to saturate the dangling bonds using halogen atoms^12^,^17^. First-principles calculations indicate that fluorinated stanene (SnF) and iodinated germanene (GeI) films are 2D TIs with sizeable nontrivial bulk gaps of about 0.3 eV at the Γ point, considerably larger than the values of the undecorated 2D systems. The halogenated germanene and stanene films that are free from dangling bonds interact weakly with substrates, making their nontrivial topologies quite robust. A question that naturally arises is: can a trivial group III-V compound film, such as GaAs, be driven to a TI with robust nontrivial topologies using the same strategy?

Here, from first-principles calculations combined with a tight-binding (TB) model, we demonstrate theoretically that under tensile strain, fluorinated GaAs film with atomic thickness becomes a TI with a large SOC gap of about 257 meV. The GaAs film is free from dangling bonds and thus chemically stable. The dynamic stability is confirmed by the phonon spectrum. Strain-induced band reversion at the Γ point is responsible for the TI phase. Using a TB Hamiltonian, we reveal that the weakening of the Ga-As σ-bonds under tensile strain dominants the band inversion and the TI phase transition. Compared with SnF and GaI films^12^,^17^, the structure inversion asymmetry in the fluorinated GaAs film leads to exotic physical phenomena, such as Rashba and Dresselhaus spin-orbital coupling. In view of the well-developed GaAs technology, our work suggests a possible route to the fabrication of QSH-based devices.

## Results

**Figure 1:**
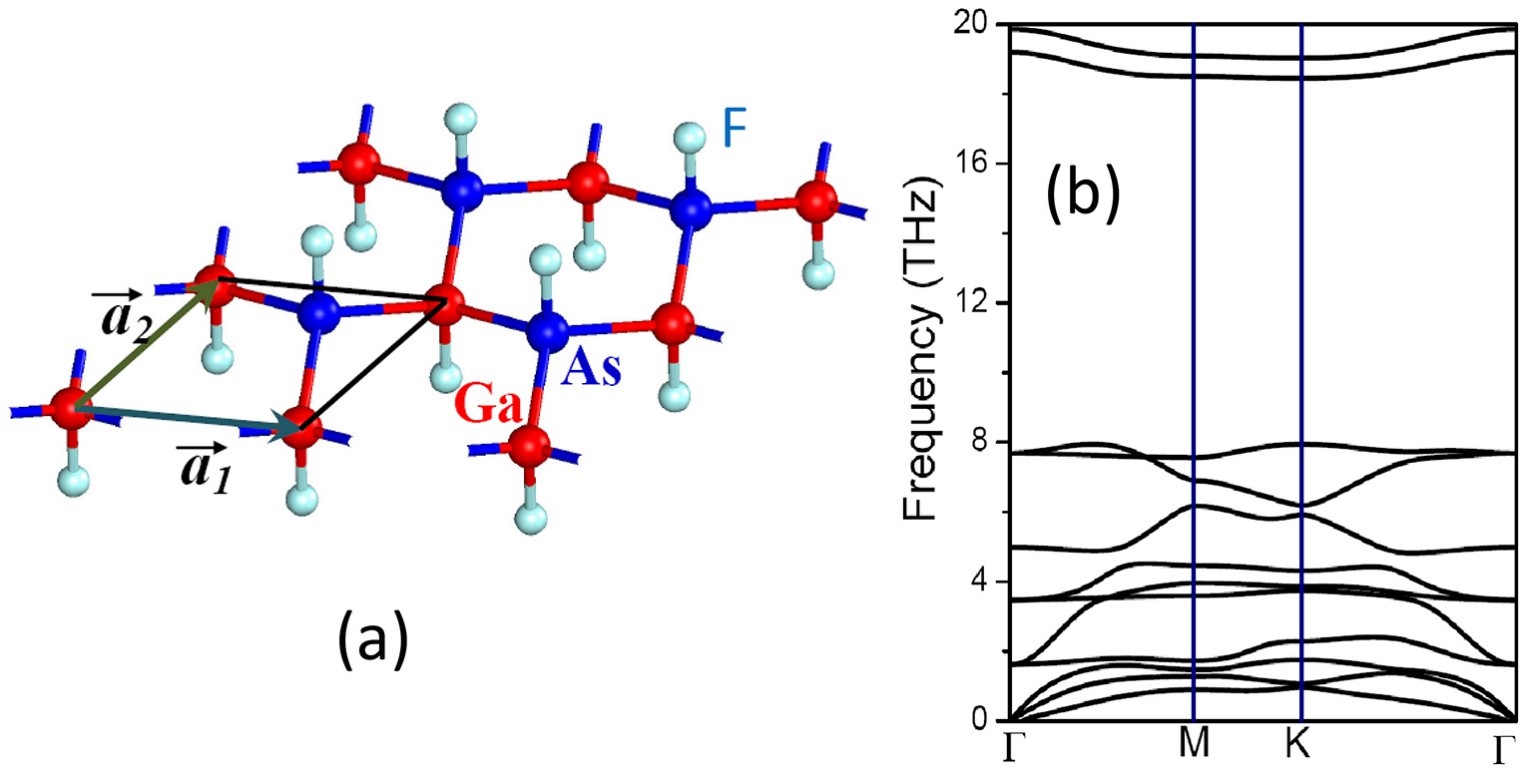
Schematic representations of the (2 × 2) supercell of a GaAs film decorated by F atoms. The unit cell is indicated by the rectangle. 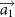 and 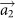 are the two base vectors. (b) Phonon spectrum of the fluorinated GaAs film along high-symmetric points in the Brillouin zone. The fractional coordinates of these points are Γ (0, 0), M(1/2, 0), and K(2/3, 1/3), respectively.

The atomic structure of GaAs film studied in this work has a buckled honeycomb lattice with each atom being passivated by F atoms, as shown in Fig. 1(a). The space group of the film is p3m1 (no.156). Both Ga and As atoms are *sp*^*3*^-hybridized and bond to four atoms (three are As or Ga, one is F), analogous to the case of the bulk counterparts. However, the T_d_ symmetry along the [111] direction of GaAs crystal is broken in this GaAs film. There is only C_3v_ symmetry in this film. The Ga-As bond length is about 2.521 Å, slightly longer than that in GaAs crystal 2.489 Å. The optimized lattice constant (the length of base vectors) is 4.226 Å. The altitude of the buckled lattice measured from the distance between the Ga-plane and As-plane is 0.633 Å. F atoms are right above (or below) the Ga (or As) atoms with the Ga-F and As-F distances of 1.776 Å and 1.781 Å, respectively. We also evaluated the binding energy between F and Ga (or As) from the difference between the total energy of half-fluorinated GaAs film and the sum of the total energies of pristine GaAs film and isolated F atom. The binding energies of the Ga-F and As-F bonds are −5.73 eV and −5.38 eV, suggesting that F atoms are chemically bonded to the GaAs film. This configuration is free from dangling bonds and thus chemically stable. The dynamic stability is confirmed by the phonon spectrum calculated along the highly symmetric directions in the BZ, as shown in Fig. 1(b). There are no modes with imaginary frequencies in the spectrum and the film is therefore expected to be dynamically stable. From the experimental point of view, the growth of GaAs ultrathin films has been achieved on the substrates, such as silicon (111) surface^18^, and fluorination of GaAs can be achieved at low temperature in CF_4_ plasma^19^. Benefiting from the well-developed GaAs technology and recent progresses in nanotechnology, the realization of the fluorinated GaAs film seems plausible in the near future.

**Figure 2:**
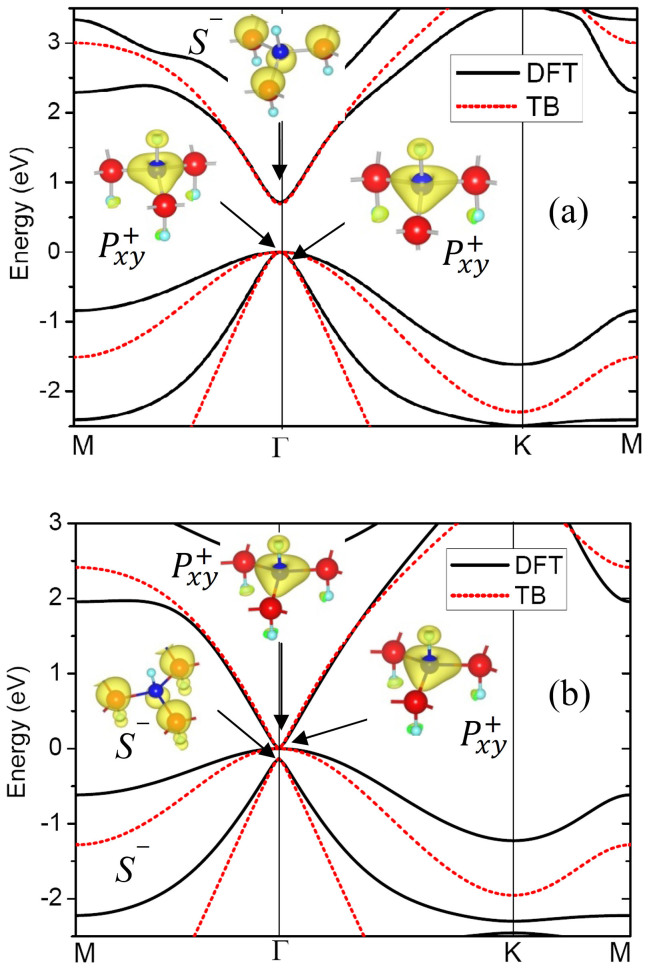
Electronic band structures of fluorinated GaAs film (a) at equilibrium state (b) under a tensile strain of 0.067. The solid lines represent the data of DFT calculations, while the band lines calculated from the TB model are indicated by the dotted lines. The electron wavefunctions of the states of valence band maximum and conduction band minimum are plotted in the insets of the figures. 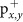 represents the binding state of *p_x,y_-*orbitals, while S^−^ is the anti-binding state of s-orbitals. The energy at the valence band maximum is set to zero.

Figure 2 gives the electronic band structures of the fluorinated GaAs film at the equilibrium state obtained from first-principles calculations without considering SOC. A trivial band gap of about 0.708 eV appears at the Γ point. The valence band maximum (VBM) is twofold degenerated and the electron wavefunctions (WFs) exhibit the features of the binding states of *p_x,y_* orbitals, whereas the WF of conduction band minimum (CBM) has the anti-binding state features of s-orbital, as shown in the insets of Fig. 2 (a). The degeneracy of the two *p_x,y_* bands at the Γ point is a direct consequence of the C_3v_ symmetry of the lattice. Fluorination moves the *p_z_* bands to the region far away from the Fermi level^12^,^17^. Obviously, at the equilibrium state, this film is a trivial semiconductor. It is noteworthy that the symmetries of the valence and conduction bands at the Γ point near the Fermi level differ significantly from bulk GaAs crystal. For the zinc blended GaAs crystal, the p-states below the Fermi level belong to a four-fold representation of Γ_8_ double group, while the conduction s states are two-fold Γ_6_. However, the fluorinated GaAs film only has a C_3v_ symmetry and the symmetries of the p-states and s-states close to the Fermi level are reduced to two-fold Γ_6_ and singlet Γ_1_.

We then try to modify the electronic structures of the fluorinated GaAs film by applying biaxial tensile strain. In our calculations, this was achieved by fixing the lattice constant (*a*) to a series of values larger than that of the equilibrium state (*a_0_*). The tensile strain is defined as *τ = (a−a_0_)/a_0_*. With the increase of the tensile strain, the band gap decreases and eventually closes as τ ≥ 0.067, as shown in Fig. 2(b). More interestingly, for the gapless GaAs film, band inversion takes place at the Γ point. The two *p_x,y_* bands touch at the Fermi level, whereas the *s* band moves down to the valence band region. Such band inversion also takes place in many other TIs^5^,^12^,^17^,^20^,^21^,^22^, but is firstly reported for III–V compound materials.

**Figure 3:**
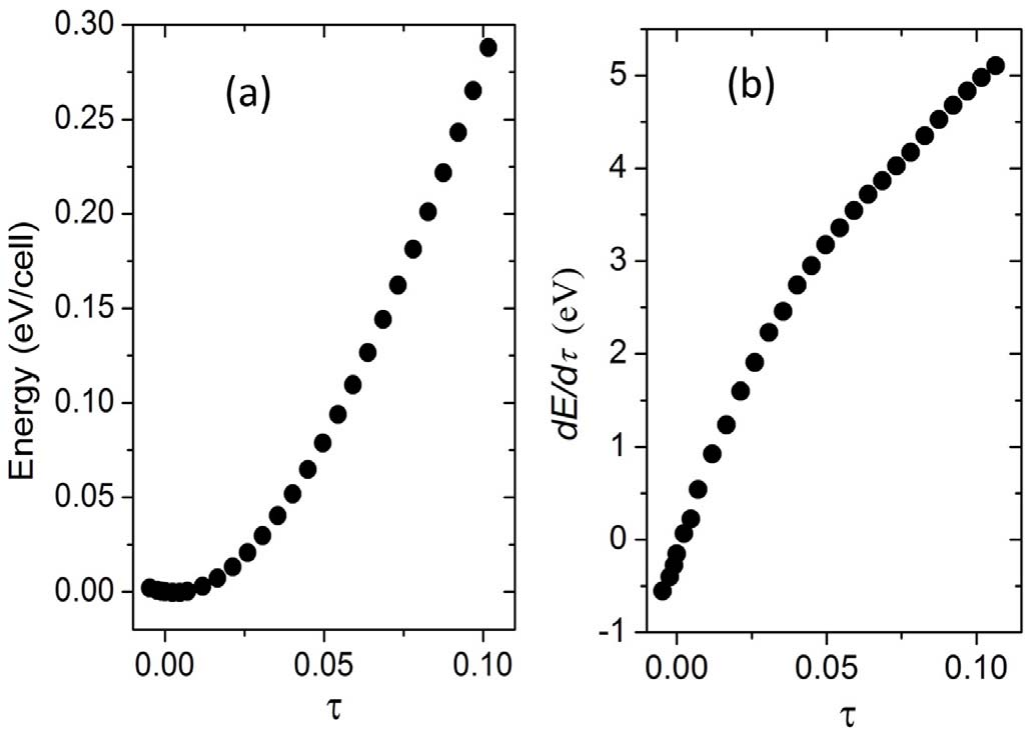
(a) Energy and (b) energy derivative with respect to the tensile strain τ. The energy at the equilibrium state is set to zero.

The stability of the fluorinated GaAs under such large tensile strain was checked. The energy and energy derivative with respect to tensile strain are plotted in Fig. 3. It is clear that with the increase of tensile strain, both energy and energy derivative increase smoothly without abrupt changes as τ < 0.1, which excludes possible structural transition and fracture. The high stability of the ultrathin film is related to the buckling configuration which can undergo large tensile strain.

To illustrate the band inversion mechanism explicitly, we propose a tight-binding model of *s*, *p_x_*, and *p_y_* orbitals. The effective Hamiltonian is taken as:
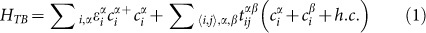
Here, 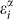, 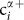, and 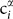 represent the on-site energy, creation, and annihilation operators of an electron at the α-orbital of the *i*-th atom. The 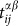 parameter is the nearest-neighbor hopping energy of an electron between an α-orbital of *i*-th atom and β-orbital of *j*-th atom, *α,β* ∈ (*s*, *p_x_*, *p_y_*). According to TB theory, the hopping energies can be evaluated using the following expressions. 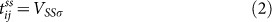

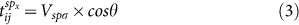

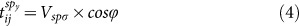

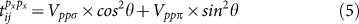

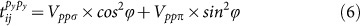

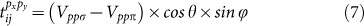


θ and φ are the angles of the vector pointed from *i*-th atom to *j*-th atom with respect to x- and y-axis. The on-site energies of *s*- and *p*-orbital are set to the values of bulk GaAs crystal, which are (−12.00 eV, −5.67 eV) for Ga and (−17.68 eV, −8.30 eV) for As^23^. Other parameters optimized at the equilibrium state are *V_ssσ_* = −1.707 eV, *V_spσ_* = 2.056 eV, *V_ppσ_*, = 2.650 eV, and *V_ppπ_* = −0.827 eV, respectively. The profile and order of the three bands nearest to the Fermi level obtained from the above TB Hamiltonian are in good agreement with the DFT results, as shown in Fig. 2(a).

When biaxial tensile strain is applied, Ga-As bond is stretched and the lattice is relaxed correspondingly. The interactions between Ga and As are therefore weakened. To reflect the strain effects, we reduce the hopping energy 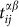 by a factor of γ (0 < γ ≤ 1), while keeping the on-site energies unchanged. The larger the tensile strain, the smaller γ is. With the decrease of γ, the band gap decreases and eventually closes as γ ≤ 0.86, as shown in Fig. 2(b). Accompanied by the closure of the band gap, *s-p*-type band inversion takes place. These results are in good agreement with those of DFT calculations. We therefore deduce that the stretching-induced weakening of Ga-As bond dominates the *s-p*-type band inversion. The strategy may also hold for the GaF and SnF films^12^,^17^. It is noteworthy that both GaF and SnF films have structure inversion symmetry, whereas the fluorinated GaAs hasn't. In this sense, fluorinated GaAs represents a more general model system of buckled honeycomb lattices. Some exotic physical phenomena arising from the breakage of inversion symmetry, such as Rashba and Dresselhaus spin-orbital coupling, appear in the GaAs film as described below.

**Figure 4:**
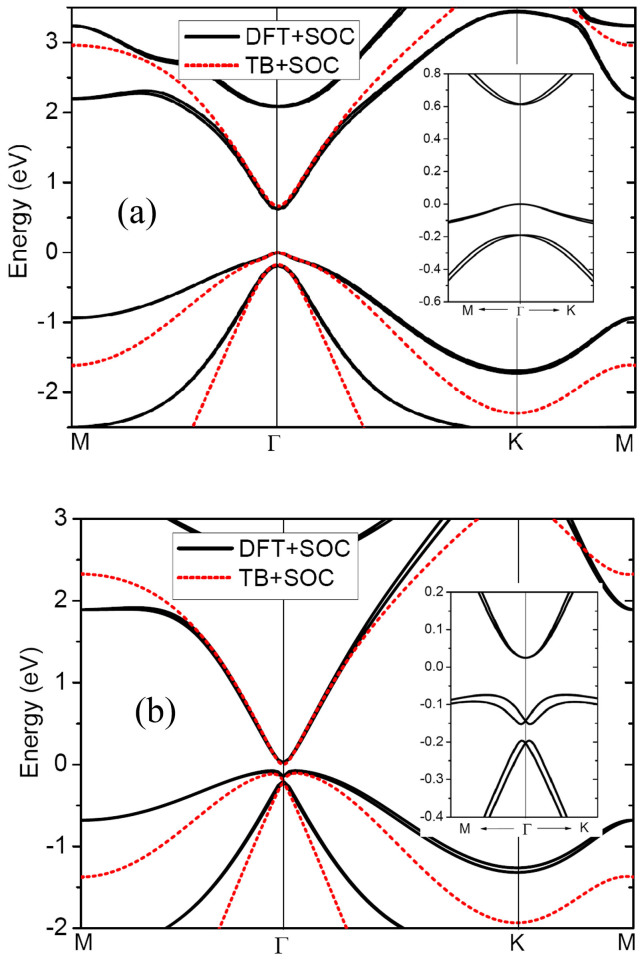
Electronic band structures of fluorinated GaAs film (a) at equilibrium state (b) under a tensile strain of 0.067. The solid lines represent the data of DFT calculations, while the band lines calculated from the TB model are indicated by the dotted lines. SOC is taken into account in both cases. The energy at the valence band maximum is set to zero. The enlarged views of the three bands nearest to the Fermi level are presented in the insets of this figure.

We then turn on the SOC in the DFT calculations. The band structures of the stretched and unstretched GaAs films are plotted in Fig. 4. Unsurprisingly, SOC opens a band gap in the gapless film, indicating that the tensile strain drives the trivial GaAs film to a TI. The band gap opened at the Γ point is about 168 meV, accompanied by an indirect band gap of 98 meV, both of which are much larger than thermal energy at room temperature (26 meV). Considering that the PBE functional may underestimate band gaps of GaAs, we adopted a more accurate Heyd-Scuseria-Ernzerhof (HSE) screened Coulomb hybrid density functional^24^ to recalculate the band gap values of the film. HSE functional gives a nontrivial band gap of about 257 meV at the Γ point. Such a large SOC gap is quite promising for achieving QSH states at room temperature. The spin degeneracy is lifted at the zone except the Γ point. Such spin-splitting has also been found in GaAs quantum wells^25^, where the lifting of spin degeneracy due to SOC leads to terms linear in electron wave vector ***k*** in the effective Hamiltonian^26^. The origin of the linear terms in low-dimensional systems is structure inversion asymmetry which lead to Rashba and Dresselhaus spin-orbital terms in the Hamiltonian^26^,^27^. The spin splitting is crucial for the field of spintronics, because it allows the electric field control of spin polarization, determines the spin relaxation rate, and can be utilized for all-electric spin injection^28^.

Our TB Hamiltonian is also available for understanding the band gap opening process by involving a spin-orbital component (*H^SO^*). 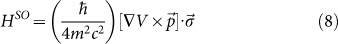
The major SOC comes from the orbits close to the atomic nuclei. Therefore, the crystal potential 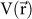 can be approximated by the spherical atomic potential. By averaging the radial degree of freedom, it reads
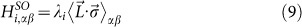

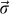
 is the vector of the of the Pauli matrices and 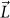 is the angular momentum operator. The matrix element<…>*_αβ_* is given in the basis of directed atomic orbitals(*α*, *β*) and *λ_i_* is the SOC strength of the *i*-th atom. The matrix elements of the dimensionless SOC operator 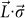 for the relevant orbitals (*s*, *p_x_*, and *p_y_*) in the 2D system are:
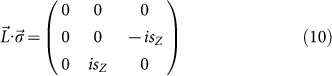
The SOC strengths of Ga and As atoms are set to 0.058 eV and 0.140 eV, respectively^28^. The electronic band structures obtained from the TB + SOC Hamiltonian for the fluorinated GaAs film with γ = 1.0 and 0.86 are plotted in Fig. 4. The TB band lines agree well with the DFT results, especially the appearance of direct band gap for γ = 1.0 and indirect band gap for γ = 0.86. This further confirms that tensile strain drives the GaAs film to a TI phase, and the band gap in the stretched film is due to the intrinsic spin-orbital coupling. The weakening of the Ga-As bonds under tensile strain is responsible for the transition from a trivial semiconductor to a TI. This mechanism still holds for other group III–V films. Unfortunately, the fine spin splitting features revealed by DFT calculations cannot be reproduced in the framework of this TB + SOC Hamiltonian due to the limitation of the H_SO_ term. More accurate Hamiltonian is therefore needed in future work.

**Figure 5:**
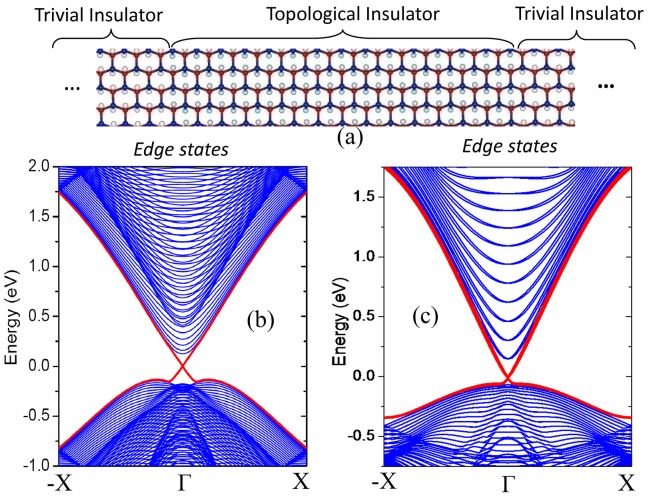
Schematic representation of the superlattice of topological insulator/trivial insulator, X = 0.5π/L. (b) Band structure of an armchair-edged nanoribbon superlattice of topological and trivial insulators. The width of the nanoribbon is about L = 43 nm. (c) Band structure of armchair nanoribbon with L = 10.9 nm, obtained by using first-principles calculations. The helical edge states are indicated by the red lines.

There are two strategies that have been widely adopted to verify the topological nontriviality of a TI. One is non-zero Z_2_ topological invariant. Another is the existence of helical gapless edge states in TI. In this work, we construct a literal superlattice of trivial insulator and TIs which are controlled by the γ values, as shown in Fig. 5(a) to study the edge states. Without loss of generality, we consider armchair-type interfaces (edges) between the two components. The widths of the trivial and nontrivial insulator nanoribbons are selected to be large enough to avoid interactions between the edge states. The calculated band structures of the superlattice using the TB + SOC Hamiltonian are presented in Fig. 5(b). One can easily see the helical edge states that form bands dispersing in the bulk gap and crossing linearly at the Γ point, which implies the topological nontriviality of the stretched GaAs film. To further confirm the existence of helical edge states, we also calculated the band structures of a stretched GaAs nanoribbon with armchair-shaped edges using first-principles calculations. The width of the nanoribbon is about 10.9 nm and the edge atoms are passivated by F atoms. The band structures of the GaAs nanoribbon are plotted in Fig. 5(c), where the helical edge states are quite obvious. The profile of the conducting edge states also agrees well that obtained from TB + SOC Hamiltonian. The Fermi velocity of the edge states is about 3 × 10^5^ m/s, which is about 1/3 of that in graphene. Helical edge states are very useful for electronics and spintronics owning to their robustness against scattering.

It should be mentioned that such strain-induced phase transition from trivial semiconductor to nontrivial topological insulator also holds for other fluorinated group III–V compound films, such as InAs. Our first-principles calculations show that the critical tensile strain of the phase transition is about 0.046 for fluorinated InAs film, slightly lower than that of the GaAs. The topological nontrivial band gap of 234 meV at the Γ point in the stretched InAs film is also comparable to that of the GaAs film. This implies the strain-induced topological nontriviality is a general feature of fluorinated group III–V compound films. Additionally, the critical tensile strain corresponding to the closure of the trivial band gap was determined by using the DFT calculations within the PBE functional, which is known to underestimate the band gap. The critical tensile strain may be thus underestimated. We therefore employed a hybrid HSE density functional^24^ to determine the critical tensile strains and found that they are about 0.11 for GaAs and 0.09 for InAs, respectively. Both films remain stable under such tensile strains.

## Discussion

The critical tensile strain of around 0.1 that triggers the trivial-semiconductor to TI phase transition is basically unrealistic for bulk GaAs materials, but accessible for ultrathin film. For example, the uniaxial and biaxial strains larger than 10% have been achieved experimentally in graphene^29^,^30^. Our simulations have demonstrated that the ultrathin GaAs film considered in this work can sustain high tensile strain (>10%) without the appearance of structural transition and bond breakage. Therefore, similar strategies may be useful for the experimental realization of high tensile strain in GaAs film.

Additionally, a recent theoretical work shows that GeI film is a TI without the need of tensile strain^17^. We therefore considered an iodinated GaAs film with structure similar to Fig. 1(a). Indeed, the iodinated GaAs film is a TI with a sizeable SOC band gap of 115 meV (PBE result) at the Γ point even at the equilibrium state. We attribute it to the hybridization of the *p_x,y_* orbitals of Ga and As atoms with the *p_x,y_* orbitals of I atom, which rises the energy of the *p_x,y_* bands, leading to the *s-p*-type band inversion. For the fluorinated GaAs film, such orbital hybridization is very weak. However, the TI phase in the iodinated GaAs film is not robust. The phonon spectrum of the iodinated GaAs film contains modes with imagery frequencies, suggesting that it is dynamically unstable. This is further confirmed by molecular dynamics simulations which show that the configuration with vertical Ga-I and As-I bonds converts to a more stable configuration with titled Ga-I and As-I bonds. For the later configuration, C_3v_ symmetry is lifted. The iodinated GaAs film becomes a trivial insulator.

It is noteworthy that substrate materials are evitable in device application. For the fluorinated GaAs film which is free from surface dangling bonds, it interacts weakly with substrates. The weak interaction won't destroy the TI states. In some special cases, the substrates may enhance SOC effect of the 2D material deposited on them. For example, the recent work of Zhou et al. showed that the hexagonal lattice of Bi atoms grown on the Si(111) surface functionalized with one-third monolayer halogen atoms exhibit isolated QSH state with an energy gap as larger as ~0.8 eV, due to a substrate-orbital-filtering effect^35^. This opened a new and exciting avenue for exploration of large-gap topological surface/interface states.

To conclude, from a tight-binding model and first-principles calculations, we demonstrate theoretically that that tensile strain can drive a trivial fluorinated GaAs film to a topological insulator with a large SOC band gap. Stretch-induced weakening of Ga-As bond is responsible for the s-p-type band inversion and the TI phase. The nontrivial bulk band gap opened due to the spin-orbital coupling (SOC) is about 257 meV, sufficiently larger for the realization of QSH effects at room temperature. This work suggests a possible route to the fabrication of QSH-based devices using the well-developed GaAs technology.

## Methods

We performed first-principles calculations within density-functional theory (DFT) using the Vienna *ab inito* simulation package (VASP)^31^,^32^. The ion-electron interactions are treated using projector-augmented-wave potentials^33^. A generalized gradient approximation (GGA) in the form of Perdew-Burke-Ernzerhof is adopted to describe the electron-electron interactions^34^. The electron wave functions are expanded using the plane waves with the energy cutoff of 600 eV. The Kohn-Sham equation is solved self-consistently with the convergence of 10^−8^. The Brillouin zone (BZ) is integrated with symmetry reduced (12 × 12 × 1) Monkhorst-Pack mesh. The system is modeled by unit cells repeated periodically on the x-y plane, while a vacuum region of about 15 Å is applied along the z-direction to exclude the interactions between images. Structural optimizations are carried out using a conjugated gradient (CG) method until the remaining force on each atom is lower than 0.001 eV/Å. The phonon spectra are calculated using a supercell approach within the PHONON code.
